# Doses of Botulinum Toxin in Cervical Dystonia: Does Ultrasound Guidance Change Injection Practices?

**DOI:** 10.3390/toxins16100439

**Published:** 2024-10-11

**Authors:** Alexandre Kreisler, Léa Mortain, Kaëlig Watel, Eugénie Mutez, Luc Defebvre, Alain Duhamel

**Affiliations:** 1 Department of Neurology and Movement Disorders, CHU Lille, F-59037 Lille, France; kaelig.watel@chu-lille.fr (K.W.); eugenie.mutez@chu-lille.fr (E.M.); luc.defebvre@chu-lille.fr (L.D.); 2 Centre d’Etude et de Recherche en Informatique Médicale, EA 2694, Université de Lille, CHU Lille, F-59045 Lille, France; mortainlea@outlook.fr (L.M.); alain.duhamel@univ-lille2.fr (A.D.); 3 LilNCog-Lille Neuroscience & Cognition, Inserm U1172, Université de Lille, CHU Lille, F-59045 Lille, France

**Keywords:** botulinum toxin, cervical dystonia, guidance, ultrasonography, long term follow-up

## Abstract

Background: Cervical dystonia is widely understood to benefit from botulinum toxin injections. The injection practices may be influenced by specific factors, including the method of injection. Three main guidance methods can be used: palpation of anatomical landmarks, ultrasound, and electromyography. We investigated how target muscles and doses of botulinum toxin were modified after the transition from surface anatomy (non-guided) to ultrasound (US-guided), in patients with cervical dystonia. We also determined the long-term dose trend. Methods: We studied a group of 82 patients, who received non-guided injections (median: 16.5 cycles/5.1 years) followed by US-guided injections (median: 12.0 cycles/3.8 years). Results: More muscles, and especially deep muscles, were injected during the US-guided period. The total dose and number of injected muscles were higher when US guidance was used, but the mean dose per muscle was lower. Over the long term, the total dose stabilized, and the mean dose per muscle decreased during the US-guided period. Conclusions: According to our results, the guidance method has a strong impact on the botulinum toxin injection strategy in cervical dystonia (target muscles and dose). Also, the treatment appeared more stable when using US guidance; this could be explained by the good precision of such injections.

## 1. Introduction

Cervical dystonia (CD) was recognized as soon as at the beginning of the 18th century [1]. With a prevalence of 28–183 cases/million, it is the most common form of focal, idiopathic dystonia [2]. In focal forms, repeated botulinum neurotoxin (BoNT) injections are definitely effective [3] and considered the first-line therapy. As the clinical presentation of CD is heterogeneous and sometimes complex [4], the choice of target muscles varies from one patient to the other and may be difficult.

Several techniques can be used to inject BoNT. Target muscles are located using palpation of anatomical landmarks (surface anatomy), electromyographic recording (EMG), or ultrasound (US). Surface anatomy-guided injections may seem easy to perform, but good knowledge of cervical anatomy is necessary. In addition, benchmarks are difficult to find in some patients. EMG is currently used in some countries [5] and provides help in the choice of target muscles [6], despite a recent meta-analysis found no evidence that EMG influences the outcome of the injections [3]. Moreover, EMG will determine if a muscle is hyperactive but not what muscle it is. Many theoretical advantages of US-guided injections have been described [7] but the superiority of US-guided injections over anatomy-guided injections was not demonstrated in all studies [8,9] and this technique was never compared to EMG-guided injections. In contrast, our group demonstrated in a cadaver study that the accuracy of US-guided injections is superior to the accuracy of anatomy-guided injections (95.3 versus 58.3% in superficial muscles; 86.3 versus 40% in deep muscles) [10]. The good accuracy of US-guided injections could have an impact on the doses of BoNT.

Only one study was designed to determine, in the long term, the course of BoNT doses in the treatment of CD [11]. Doses increased (from 180 ± 65 U to 203 ± 65 U by cycle) when the old formulation of onabotulinumtoxin A (batch 7911) was used, but not with the new formulation (batch 8804). The follow-up was for 5 years with each formulation. Other studies provided various results. Hsiung et al. [12] found no significant increase. In three studies, the doses apparently increased but no statistical analysis was performed [13,14,15]. In three other studies, the doses increased in a group of various movement disorders including CD, but no analysis was performed in the CD subgroup [16,17,18]. In Kessler et al.’s study [19], the dose seemed stable after a first, booster dose; however, no statistical analysis was performed. In Jochim et al.’s study [20], doses increased during the first five years of treatment and then leveled off. Lastly, Sen et al. [21] found an increase in doses but also the number of injected muscles. The results of all such studies are summarized in Table 1 [11,12,13,14,15,16,17,18,19,20,21,22,23,24,25,26,27,28,29,30,31,32].

The main objective of the present study was to determine how, in daily practice, the target muscles and doses of BoNT in patients with CD were modified after a change in the method of injection, from surface anatomy to US. The secondary objective was to specify the course of BoNT doses over time in the long term, according to the injection method.

## 2. Results

Eighty-two patients were included in the US group (non-guided injections followed by US-guided injections), and 48 patients were included in the No US group (non-guided injections only). We studied 2575 injection cycles in the US group (1652 were non-guided and 923 were US-guided) and 1658 injections cycles in the No US group. Demographic data are summarized in Table 2.

Some patients were not included for the following reasons: incomplete data (five patients; the first injections were performed in other hospitals); lost to follow up (one patient); target muscles dramatically changed after the switch (two patients with an antecaput—non-guided: sternocleidomastoid; US-guided: longus collis); and opposition to the study (one patient).

In the US group, ten patients (12.2%) received abobotulinumtoxin A only, 36 patients (43.9%) received onabotulinumtoxin A only, 1 patient (1.2%) received incobotulinumtoxin A only, and 35 patients (42.7%) received the three toxins. In the No US group, 4 patients (8.3%) received abobotulinumtoxin A only, 37 patients (77.1%) received onabotulinumtoxin A only, 1 patient (2.1%) received incobotulinumtoxin A only, and 6 patients (12.5%) received the three toxins.

Table 3 and Table 4 summarize, respectively, the number of injection cycles and the frequency of injections in each group. In the US group, some muscles were injected more frequently after switching to US guidance: trapezius, levator scapulae, semispinalis capitis, longissimus capitis, scalenus anterior, obliquus capitis inferior, and semispinalis cervicis. The frequency of injections in the splenius capitis also varied significantly, despite the percentages looking similar in the two groups. A more precise analysis indicated that this muscle was injected more frequently during the first period of time (non-guided injections) in a subgroup of only twelve patients, which explains the lack of impact on the percentages. Percentages were similar for three other muscles, because such muscles were rarely injected whatever the method: longissimus capitis (non-guided: 0 patients; US-guided: 19 patients (92 cycles)), scalenus anterior (non-guided: 2 patients (9 cycles); US-guided: 11 patients (70 cycles)), semispinalis cervicis (non-guided: 6 patients (7 cycles); US-guided: 24 patients (170 cycles)). In the No US group, the sternocleidomastoid, splenius capitis, and levator scapulae were injected more frequently than in the US group (considering non-guided injections only). Similar results were found regardless of the conversion ratio between two toxins.

Table 5 summarizes the doses injected in each muscle, the total dose, the mean dose per muscle (total dose divided by the number of injected muscles), and the number of injected muscles (per cycle) (conversion ratio 2.5:1). In the US group, the total dose and the number of injected muscles were higher when US guidance was used, but the mean dose per muscle was lower. The dose was lower in the splenius capitis and in the sternocleidomastoid when US guidance was used, but higher in the trapezius and in the levator scapulae. In the No US group, the dose in the splenius capitis was higher and the dose in the levator scapulae was lower than in the US group (considering non-guided injections only). Similar results were found regardless of the conversion ratio (results with the 3:1 conversion ratio are presented as Supplementary Materials, Table S1).

Table 6 summarizes the BoNT dose at the first and last injection cycles with each technique (conversion ratio 2.5:1). Similar results were found regardless of the conversion ratio (results with the 3:1 conversion ratio are presented as Supplementary Materials, Table S2).

**Figure 1:**
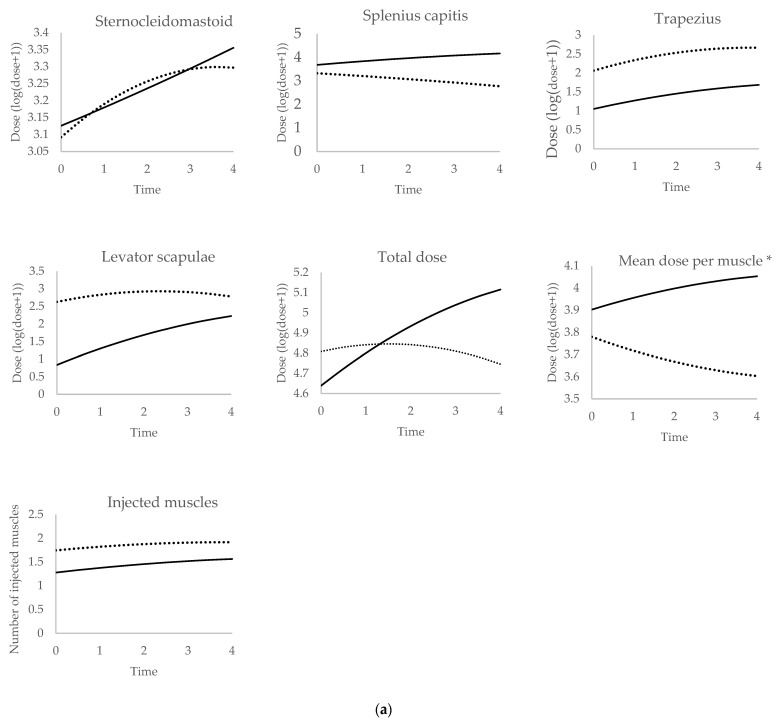

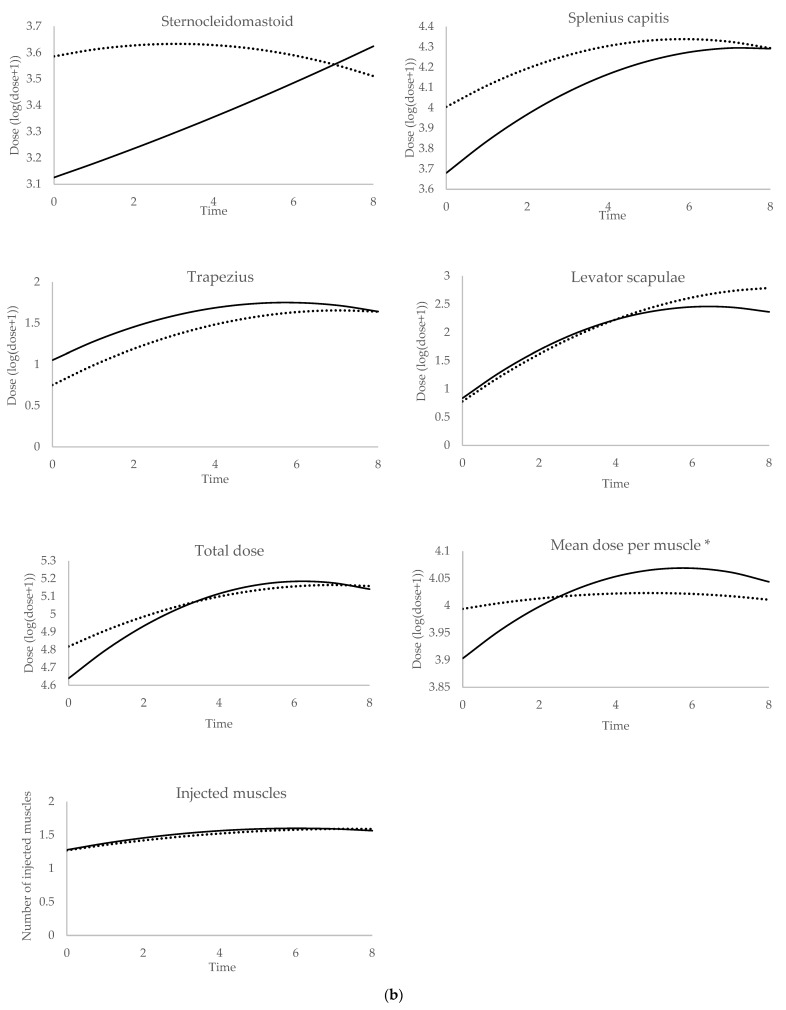
(**a**) Changes in botulinum neurotoxin doses over time according to each injection method in the US group (conversion ratio abobotulinumtoxin A:onabotulinumtoxin A 3.0:1.0). Full lines: US group, non-guided injections. Doted lines: US group, US-guided injections. Dose = log (number of onabotulinumtoxin A units + 1). The time is expressed in years. Time 0 is the first day of injections. * Total dose / number of injected muscles. (**b**) Changes in botulinum neurotoxin doses over time according to the group, in the absence of guidance (conversion ratio abobotulinumtoxin A:onabotulinumtoxin A 3.0:1.0). Full lines: US group, non-guided injections. Doted lines: No US group. Dose = log (number of onabotulinumtoxin A units + 1). The time is expressed in years. Time 0 is the first day of injections. * Total dose / number of injected muscles.

Figure 1a shows the changes in botulinum neurotoxin doses over time according to each injection method in the US group (non-guided and US-guided). Figure 1b shows the changes in botulinum neurotoxin doses over time according to the group (US group and No US group), in the absence of guidance. We only present the results obtained with the conversion ratio abobotulinumtoxin A:onabotulinumtoxin A 3:1; the curves with the 2.5:1 ratio are very similar.

Table 7 summarizes our comparisons of long-term dose changes (mixed procedure): non-guided period versus US-guided period in the US group; and non-guided period in the US group versus the No US group (conversion ratio 2.5:1). Similar results were found regardless of the conversion ratio (results with the 3:1 conversion ratio are presented as Supplementary Materials, Table S3). In the US group, doses increased throughout the follow-up when non-guided injections were used, regardless of the muscle; the total dose, the mean dose per muscle, and the number of target muscles also increased. After switching from non-guided to US-guided injections, some changes were observed: the dose in the splenius capitis, the dose in the levator scapulae, and the total dose were more stable; and the mean dose per muscle decreased. The dose in the trapezius and the number of target muscles increased similarly in both groups. We found no difference between the No US group and the US group (considering non-guided injections only). Only a tendency could be observed for the total dose and the mean dose per muscle, which seemed more stable in the No US group. Regarding the sternocleidomastoid, comparisons were not performed because we could not match the curves with a specific model; i.e., the curve was neither linear (following a straight line) nor quadratic (graphed as a parabola).

## 3. Discussion

In the first part of this study, we studied a group of 82 patients who received BoNT injections for cervical dystonia with two injection methods. During the first period, the injections were based on surface anatomy (non-guided); and during the second period, injections were US-guided. We compared the doses of BoNT and the target muscles according to each injection period; in the long term, the median number of injection cycles was 28.0 (Table 2). To the best of our knowledge, this is the first comparison of a non-guided period to a US-guided period. We found different strategies of injection according to the guidance method. As expected, most deep muscles were injected more frequently when US guidance was used. Surprisingly, the splenius capitis was injected less frequently during the US guidance period. Indeed, in twelve patients, the injections stopped in the splenius capitis (we assume a lack of efficiency) after switching to US guidance, and other muscles were targeted: OCI alone (four patients), OCI and semispinalis capitis (four patients), semispinalis capitis alone, and semispinalis cervicis, levator scapulae, or scalenus anterior (one patient each). As another unexpected result, the trapezius and the levator scapulae were injected more frequently during the US period. We suggest two explanations. Firstly, it may be difficult to target such muscles without guidance, especially when they atrophy after repeated injections of BoNT. Secondly, the promotion of the so-called “colli caput concept” could have brought such muscles up to date. This is especially true for the levator scapulae. This muscle is subdivided into several distinct parts from origin (transverse processes of C1, C2, C3, and sometimes C4) to insertion (scapula). It was suggested that US-guided specific injections of C1 on the one side, or C2–C4 on the other side, do not have the same effect (on caput dystonic subtypes and colli dystonic subtypes respectively) [33], while it seems difficult to target specific muscle bundles without guidance.

We found that more muscles were injected in each injection cycle when US guidance was used (median: 5.0 versus 3.0). This is probably the consequence of the easier access to deep muscles with sonography. The total dose by cycle decreased with US guidance (Table 5). In parallel, the mean dose per muscle decreased after switching to US (Table 5). We can consider two different explanations. The main hypothesis is that the physician reduced the dose in each muscle as the total number of target muscles increased, aiming to lessen the risk of side effects (mainly dropping head). As another possibility, the good precision improved the outcome and enabled the physician to reduce BoNT doses. Curves showing dose changes over time in the long term (Figure 1a) support the second hypothesis as the mean dose per muscle decreased during the US guidance period. The stabilization of the dose in the splenius capitis and levator scapulae, as well as the total dose, may also indicate that US-guided injections allow a more reproducible outcome.

In the second part of this study, we studied a group of patients in whom US guidance had not been used, at the physician’s choice (“No US group”). In the “US group”, the doses injected during the non-guided period may not reflect what happens in all patients with CD: the need to use US in a second step indicates a more severe form of the disease, or more demanding patients. That is why we compared the “No US group” to the non-guided period in the “US group”. If the number of target muscles and the mean dose per muscle were not different, the median dose in the splenius capitis and in the levator scapulae were higher in the “No US group” (Table 5). Moreover, the SCM, splenius capitis and levator scapulae muscles were injected more commonly in the “No US group” (Table 4). Consequently, we cannot exclude that, with higher BoNT doses in some muscles, US-guided injections would not have been necessary in some of the patients.

In the last part of the study, we studied the change in BoNT doses over time in the long term (in the US group and the No US group). The follow-up was one of the longest in the literature (Table 1 and Table 2). In CD patients, BoNT dose changes have been described in ten studies (Table 1). Statistical analysis were performed in four of them, providing controversial results: the dose was stable in two studies [11,12] (Hsiung 2002; Garcia-Ruiz, 2011) and increased in the two others [20,21] (Sen, 2014; Jochim, 2019). Garcia-Ruiz et al. studied two batches of onabotulinum A: the old batch (before 2000) and the new batch (after 2000) [11]. The dose increased with the old batch and remained stable with the new batch. Jochim et al. found a moderate increase in the first five years; then, the dose remained stable (when, in the present study, the dose increased continuously; Figure 1) [20]. The difference between studies could be explained by different habits from one physician to the next. Among the results of our study, only those obtained without US guidance can be compared with data from the literature. A special feature of our work was to study not only the total dose but also the dose in four specific muscles. We demonstrated a progressive increase in the total dose. This finding is not entirely explained by an increase in the number of target muscles per injection cycle. Actually, the dose increased in three out of four of the muscles we studied: splenius capitis, trapezius, and levator scapulae. In the absence of US guidance, BoNT dose changes over time were similar in the US group and the No US group (Table 7).

During the period of US guidance, neither the total dose nor the dose in the splenius capitis and levator scapulae increased. Only the dose in the trapezius increased. An analysis with the sternocleidomastoid was not possible. The most likely explanation for this stabilization is that the doses had already increased during the non-guided period. However, it is also possible that US guidance provided a better result and put an end to dosage increases. We do not have a quantitative evaluation of the outcome of the injections to support this hypothesis.

In order to offer practical advice, we calculated the median dose at the beginning of the management and at the end of this study. According to our results, in the absence of US guidance, the optimal, standardized dose (the dose finally used to optimize the outcome and avoid side effects) was close to 50 units in the sternocleidomastoid 80 units in the splenius capitis, and 40 units in the trapezius and the levator scapulae (Table 6). The results were similar when US guidance was used; the main difference was a lower dose in the splenius capitis (40 U).

We are aware that our study had several limitations. A first pitfall was that various brands of BoNT were used. This is an important concern as, in 32.9% of the patients, the shift from one BoNT brand to another was made simultaneously with the change in guidance method. Actually, there is controversy about the dose ratio between abobotulinumtoxin A and onabotulinumtoxin A [34]. We chose to use two different but commonly admitted ratios (2.5:1 and 3.0:1). However, the ratio did not have a significant impact on our results. A second limitation is the retrospective design. Interesting data are missing, such as the clinical outcome. Other data were incomplete or unclear, such as the nature and duration of side effects. For example, these data could indicate whether the dose and method of injection have an impact on swallowing difficulties, as this is controversial in the literature [8,9,35]. It would also be interesting to study a group of patients who received US-guided injections from the start, that is, without an initial non-guided period. Lastly, in the US group, the baseline (dose per muscle; target muscles) was not the same at the start of the treatment (non-guided) and when switching to US guidance. This indicates that the differences observed between the two periods are not only a consequence of the method of injection.

## 4. Materials and Methods

### 4.1. Ethics

This study was performed in accordance with the ethical standards detailed in the Declaration of Helsinki. Moreover, this study was registered with the French National Data Protection Commission (Commission Nationale de l’Informatique et des Libertés; reference: DEC20-058). Written, informed consent was obtained from all the study participants.

We retrospectively examined the medical records of consecutive patients treated with BoNT type A (BoNT-A) injections for CD in the Movement Disorders Department at Lille University Medical Center, between January 1995 and November 2021.

### 4.2. Inclusion and Non-Inclusion Criteria

Two groups of patients were studied: the US group and the No US group. A patient was included in the US group if they had received at least three consecutive non-guided BoNT-A injections (target muscles were localized by using surface anatomy only), followed by at least three consecutive US-guided injections. A patient was included in the No US group if they had received at least six consecutive non-guided BoNT-A injections and if US-guided injections had not been considered or performed in the long term. Other inclusion criteria were (in the two groups) regular follow-up with at least three injections per year, and complete data for each injection.

### 4.3. Data

The medical files were selected from an initial file of 182 patients with cervical dystonia followed in our department.

The following data were collected: date of birth; etiology and topography of the dystonia; age at onset; main form of the dystonia (according to the col-cap concept [36]); date of each injection cycle; target muscles in each injection cycle; brand and dose of BoNT in each target muscle; and reason for using US-guided injections rather than non-guided injections.

### 4.4. Statistical Analysis

Data were analyzed using the SAS software version 9.4 (SAS Institute, Cary, NC, USA).

Categorical variables are reported as absolute numbers and percentages, whereas continuous variables are expressed as the median with interquartile range (25th–75th percentile). Statistical testing was conducted at the two-tailed α-level of 0.05.

The BoNT dose was calculated in onabotulinumtoxin A units, since this was used for the majority of the injections. We always considered incobotulinumtoxin A:onabotulinumtoxin A 1:1. According to the controversy about the conversion ratio between abobotulinumtoxin A and onabotulinumtoxin A, two different ratios were used for statistical analysis: abobotulinumtoxin A:onabotulinumtoxin A 2.5:1 and abobotulinumtoxin A:onabotulinumtoxin A 3.0:1.

For statistical comparisons, we studied the doses in the following muscles: sternocleidomastoid, splenius capitis, levator scapulae, and trapezius. The doses were not studied in other muscles as such muscles were rarely injected when non-guided injections were performed. The “total dose” was the sum of the doses in the four main muscles: sternocleidomastoid, splenius capitis, trapezius, and levator scapulae. The “mean dose per muscle” was calculated for each injection cycle: total dose (all muscles) divided by the number of target muscles during the same injection cycle.

In the US group, the comparisons between non-guided and guided injection data (number of injections and dose per muscle) were performed using the linear mixed model. To take into account the correlation between the repeated measures within each type of guidance as well as between the measures performed on the same patient according to the two types of guidance, we introduced as random effects the interaction “patient × guidance method” and the “patient”.

For the comparison between the non-guided US group and No US group, the random effect was only a patient effect, allowing us to take into account repeated measurements in each unpaired group.

In the US group, injection frequencies for each muscle were compared according to the two guidance methods using the Wilcoxon paired signed rank test. For comparisons between the non-guided US group and No US group, we used the Mann–Whitney test.

We further analyzed the change over time for the number of target muscles, dose in all muscles, and dose in each of the four muscles (sternocleidomastoid, splenius capitis, levator scapulae, and trapezius) according to the type of guidance method in the US group using a mixed model for longitudinal data, namely a random coefficients model. In this model, the origin of the time scale was the day of the first injection for the non-guided group and the day of change in method of injection for the US-guided group. Comparisons of the change over time for each parameter between the types of guidance (non-guided and guided) were made by including, as fixed effects, a linear and quadratic time effect as well as the interactions between these effects of time and the type of guidance. We used the contrast linear and quadratic derived from the model to compare the global evolution according to the type of guidance. To take into account the correlation between the repeated measures within each type of guidance as well as between the measures performed on the same patient according to the two types of guidance, we introduced as random effects an intercept, a slope, and a quadratic coefficient at the level of patient × guidance, and a random intercept at the patient level. For each parameter, the change over time was plotted according to the condition of guided or non-guided using the parameters estimated from the longitudinal linear mixed model.

To compare the changes over time for all the previous parameters between the No US group and the non-guided injections of the US group, a linear mixed model was also used. In this model, the fixed effects were the type of group (No US/US non-guided), a linear and quadratic time effect, as well as the interactions between these effects of time and the type of group. We used the contrast linear and quadratic derived from the model to compare the global evolution according to the type of group. The correlations between the repeated measurements were handed by introducing, as random effects, an intercept, a slope, and a quadratic coefficient at the patient level.

All the models were performed after log transformation of data (Log (value +1)) to normalize the model residuals.

## Figures and Tables

**Table 1:** Long-term treatment with BoNT. Summary of the literature.

Study	Main Objective	Number of Patients with CD	Follow-Up	BoNT	Mean Dose	Course of Doses
Kessler et al. 1999 [19]	Efficacy and safety in CD	303	From 2 to 6 years (6 to 21 injections)	Abo	778 ± 253 U	Starting dose: 1072 ± 373 U According to Figure 2B in [19], the dose reduced after a booster dose, then increased again after 10 cycles No statistical evaluation of dose increase was performed
Hsiung et al., 2002 [12]	Outcome in various dystonic conditions and hemifacial spasm	106	More than 5 years	Ona	222 U [70–400] (mean [range])	Dose at year 5 was superior to at year 1 and years 2 to 4 (Figure 1 in [12]), but the change was not significant
Hausserman et al., 2004 [22]	Adherence to treatment, perception of improvement and reasons for discontinuation in CD	100	61.02 ± 54.53 months (13.82 ± 14.33 cycles)	Abo	800.79 ± 241 U	No data
Skogseid et al., 2005 [23]	Efficacy in idiopathic CD	78	More than 1.5 years	Ona	111 U [82–190] (median [range])	No data
Mejia et al., 2005 [16]	Efficacy and safety in CD	19	From 12 to 18.9 years (32.4 ± 17.0 cycles)	Ona	No data	No data
Brin et al., 2008 [13]	Rate of clinical non-responsiveness and BoNT-A neutralizing Ab formation in CD	326	From 0.26 to 4.2 years (1 to 15 cycles)	Ona	187.0 ± 76.5 U	Treatment 1: 148 U (326 subjects) Treatment 12: 213 U (44 subjects) Dose increase was attributed to initial titration No statistical evaluation of dose increase was performed
Mohammadi et al., 2009 [24]	Efficacy and safety in CD	207	Ona: 5.2 ± 2.2 years Abo: 7.3 ± 3.1 years	Abo (163 patients) Ona (44 patients)	Abo: 389 ± 144 U Ona: 145 ± 44 U	No data
Maia et al., 2010 [25]	To determine if a change in the clinical pattern of CD over time is associated with a worse response to BoNT therapy	67	Stable pattern: 80 months (15 cycles) Pattern change: 62 months (11 cycles)	Ona	Stable pattern: 219.17 U Pattern change: 204.78 U	No data
Truong et al., 2010 [26]	Efficacy and safety in CD	55	From 3.9 to 94.0 weeks (1 to 4 cycles)	Abo	No data	Cycle 1: 502.3 U (108 subjects) Cycle 2: 642.5 U (100 subjects) Cycle 3: 716.1 U (96 subjects) Cycle 4: 775.6 U (88 subjects) No statistical evaluation of dose increase was performed
Camargo et al., 2011 [27]	Efficacy and safety in CD	28	No data	Abo Ona Prosigne^®^	No data	No data
Garcia Ruiz et al. 2011 [11]	Mean dose of BoNT-A per session and appearance of resistance during the first 5 years of treatment in CD	275	5 years	Ona	No data	First injected before 2000 (old batch): significant increase (from 180 ± 65 to 203 ± 63 U) First injected after 2000 (new, current batch): no increase (181+/−75 U)
Vivancos et al., 2012 [28]	Efficacy and safety in CD	37	From 1 to 17 years	Abo	487 ± 55 U	No data
Dressler et al., 2013 [29]	Efficacy and safety in CD	64 (including a maximum of 19 BoNT-naïve patients)	From 49.3 to 114.1 weeks (5 cycles)	Inco	No data	First injection: 151.4 ± 57.8 U Fifth injection: 192.2 ± 69.1 U No statistical evaluation of dose increase was performed
Gill et al., 2013 [14]	Clinical characteristics of CD patients who continued care with BoNT, and reasons for stopping care	70 (including 65 patients treated at least once with BoNT-A)	From 0 to 39 cycles (median: 14)	Ona (97% of the patients at treatment initiation) Rima Abo	Ona: 267.2 ± 81.4 U Rima: 12,770 ± 5228 U	In patients who continued care, the starting dose was 230.8 ± 74.3 U of Ona Successive doses appear in Figures 1 and 2 in [14], but no statistical evaluation of dose increase was performed
Ramirez-Castaneda and Jankovic, 2014 [17] (continuation of Mejia et al., 2005)	Efficacy and safety in various dystonic conditions	51	From 10 to 26 years	Ona (95% of the patients) Rima (3.1% of the patients) Abo (0.44% of the patients) Inco (0.12% of the patients) Clinical trial product (1.02% of the patients)	Ona: 266.18 ± 103.71 U	No specific data for CD
Sen et al., 2014 [21]	Clinical characteristics and efficacy in CD	45	From 12 to 120 months (mean: 36.13 ± 29.17)	Abo (44 patients) Ona (1 patient)	Abo: 643.23 ± 154.01 U	First injection: 599.00 ± 147.60 Last injection: 681.66 ± 188.09 U Significant increase
Jog et al., 2016 [30]	Benefit of Ona on quality of life of patients with CD	234	5 cycles	Ona	No data	No data
Bentivoglio et al., 2017 [15]	Efficacy and safety in primary CD	39	From 2 to 6 years (6 to 40 cycles)	Abo	701.5 ± 280.6	First injection: 492.6 ± 275.9 U 20th injection: 806.3 ± 197.5 (18 patients) Successive doses appear in Figure 3 in [15], but no statistical evaluation of dose increase was performed
Moll et al., 2018 [31]	Quality of life in CD	211	From 2 to 21.5 years (mean: 11.7 ± 5.3 years)	Abo only (128 patients) Ona only (36 patients) Rima (1 patient) Several toxins (Abo, Ona, Inco) (46 patients)	Abo only: 702 ± 133 U Ona only: 187 ± 32 U Rima only: 7500 U Inco: 232 ± 45 U	No data
Colossimo et al., 2019 [32]	Satisfaction of patients with CD	995 patients at baseline; 583 patients completed the survey	34.2 ± 9.9 months (8.65 ± 3.25 cycles)	Abo (689 patients) Ona (247 patients) Inco (59 patients)	Abo: 500.0 [50.0-1833.3] Ona: 150.0 [13.3–500.0] Inco: 198.6 [45.6–514.3] (Median [range])	No data
	Current real-life treatment of CD	334	At least 3 injections Abo: 11.0 ± 7.7 years Ona: 5.9 ± 5.0 years	Abo (209 patients) Ona (135 patients)	Abo: 663 ± 249 U Ona: 138 ± 51 U	Abo: First year: 585 ± 254 Fifth year: 648 ± 240 Ona: First year: 124 ± 54 Fifth year: 144 ± 55 Significant increase with both toxins
Jochim et al., 2019 [20]	Incidence and prevalence of neutralizing antibodies’ formation under Inco in various dystonic and spastic conditions, and hemifacial spasm	73	All the patients: 2149 ± 1225 days Unknown in the CD subgroup	Inco only Ona then Inco Abo then Inco	Unknown	Initial dose about 200 U (Figure 1a in [20]) Further doses unknown
Hefter et al., 2020 [18]	Incidence and prevalence of neutralizing antibodies formation under Inco in various dystonic and spastic conditions, and hemifacial spasm.	73	All the patients: 2149+/-1225 days. Unknown in the CD subgroup.	Inco only Ona then Inco Abo then Inco	Unknown	Inital dose about 200 U (Figure 1a in [18]) Further doses unknown.

Ab: antibody; Abo: abobotulinumtoxin A (Dysport^®^); BoNT: botulinum neurotoxin; CD: cervical dystonia; Inco: incobotulinumtoxin A (Xeomin^®^); Ona: onabotulinumtoxin A (Botox^®^); Rima: rimabotulinumtoxin B (Myobloc^®^ or Neurobloc^®^); U: units. All quantitative data are quoted as the mean ± standard deviation (unless otherwise indicated).

**Table 2:** Demographic characteristics in the two groups.

		US Group (*n* = 82)	No US Group (*n* = 48)
Gender	Women	58 (70.3%)	31 (64.6%)
	Men	24 (29.7%)	17 (35.4%)
Time between onset of symptoms and first injection (median (Q1; Q3))		1.89 years (0.7; 7.6)	2.96 years (1.2; 8.6)
Age at first injection (median (Q1; Q3))		51.00 years (45.0; 62.0)	48.23 years (42.5; 57.0)
Etiology of the dystonia	Idiopathic	74 (90.2%)	46 (95.8%)
	Iatrogenic	2 (2.4%)	2 (4.2%)
	Degenerative neurologic disorder	4 (4.9%)	0
	Post-traumatic	1 (1.2%)	0
	Mitochondrial cytopathy	1 (1.2%)	0
Main dystonic subtype	Torticaput	52 (63.4%)	24 (50.0%)
	Torticollis	5 (6.1%)	4 (8.3%)
	Laterocaput	18 (22.0%)	13 (27.1%)
	Laterocollis	2 (2.4)	2 (4.2%)
	Antecaput	0	0
	Antecollis	0	0
	Retrocaput	5 (6.1%)	5 (10.4%)
	Retrocollis	0	1 (2.1%)
Number of injection cycles (median (Q1;Q3))	All	28.0 (19.0;43.0)	31.5 (19.5;45.0)
	Non-guided	16.5 (8.0;30.0)	31.5 (19.5;45.0)
	US-guided	12.0 (7.0;15.0)	0.00 (0.00;0.00)
Follow-up (years) (median (Q1;Q3))	All	10.0 (6.5;14.6)	10.8 (7.6;14.0)
	Non-guided	5.1 (2.7;10.6)	10.8 (7.6;14.0)
	US-guided	3.8 (2.3;4.7)	0.00 (0.00;0.00)
Reason for switching from non-guided to US-guided injections	Low efficacy on posture	59 (72.0%)	-
	Low efficacy on tremor	20 (24.4%)	-
	Difficulty to find anatomical landmarks (for example, obesity)	3 (3.7%)	-

Q1: first quartile; Q3: third quartile; US: ultrasound.

**Table 3:** Number of injection cycles into each muscle according to the technique of injection. The US group denotes the patients for whom two techniques of injection were used (at least three non-guided injection cycles followed by at least three US-guided injection cycles). The No US group denotes the patients for whom US guidance was never used (at least six non-guided injection cycles).

Muscle	US Group		No US Group
	Non-Guided Injections	US-Guided Injections	
Sternocleidomastoid	12.0 (6.0; 25.0) 17.70 ± 15.48	10.0 (6.0; 14.0) 9.93 ± 5.62	31.00 (17.00; 45.00) 31.48 ± 20.09
Splenius capitis	15.0 (7.0; 28.0) 19.02 ± 15.32	8.0 (5.0; 13.0) 8.79 ± 5.98	31.50 (19.50; 43.50) 33.88 ± 18.88
Trapezius	5.0 (1.0; 9.0) 7.23 ± 8.02	7.0 (2.0; 13.0) 7.85 ± 6.61	5.50 (0.00; 18.50) 10.83 ± 11.75
Levator scapulae	6.0 (2.0; 13.0) 9.40 ± 11.20	7.0 (4.0; 13.0) 8.55 ± 6.20	19.50 (10.00; 26.50) 19.88 ± 14.62
Semispinalis capitis	0.0 (0.0; 2.0) 1.87 ± 3.67	4.0 (0.0; 10.0) 5.35 ± 5.54	0.00 (0.00; 0.00) 2.96 ± 6.28
Longissimus capitis	0.0 (0.0; 0.0) 0.00 ± 0.00	0.0 (0.0; 0.0) 1.12 ± 2.80	0.00 (0.00; 0.00) 0.00 ± 0.00
Scalenus anterior	0.0 (0.0; 0.0) 0.11 ± 0.89	0.0 (0.0; 0.0) 0.88 ± 2.70	0.00 (0.00; 0.00) 0.00 ± 0.00
Scalenus medius	0.0 (0.0; 0.0) 0.62 ± 2.00	0.0 (0.0; 0.0) 0.85 ± 2.66	0.00 (0.00; 0.00) 1.92 ± 5.55
Obliquus capitis anterior	0.0 (0.0; 0.0) 0.02 ± 0.16	4.5 (0.0; 14.0) 7.01 ± 7.40	0.00 (0.00; 0.00) 0.10 ± 0.59
Rectus capitis major	0.0 (0.0; 0.0) 0.00 ± 0.00	0.0 (0.0; 0.0) 0.12 ± 0.84	0.00 (0.00; 0.00) 0.00 ± 0.00
Semispinalis cervicis	0.0 (0.0; 0.0) 0.09 ± 0.32	0.0 (0.0; 1.0) 2.07 ± 4.58	0.00 (0.00; 0.00) 0.10 ± 0.37

Results are presented as median (Q1; Q3) and mean ± standard deviation. Q1: first quartile; Q3: third quartile; US: ultrasound.

**Table 4:** Frequency of injection cycles into each muscle according to the technique of injection. The US group denotes the patients for whom two techniques of injection were used (at least three non-guided injection cycles followed by at least three US-guided injection cycles). The No US group denotes the patients for whom US guidance was never used (at least six non-guided injection cycles).

Muscle	US Group (*n* = 82)			No US Group (*n* = 48)	*p* ** (Wilcoxon Test)
	Non-Guided Injections	US-Guided Injections	*p* * (Wilcoxon Test)		
Sternocleidomastoid	1.00 (0.87; 1.00) 0.86 ± 0.27	1.00 (0.91; 1.00) 0.87 ± 0.30	0.17	1.0 (0.98; 1.00) 0.90 ± 0.25	0.027
Splenius capitis	1.00 (0.95; 1.00) 0.93 ± 0.19	1.00 (0.76; 1.00) 0.80 ± 0.35	0.0237	1.0 (1.00; 1.00) 0.99 ± 0.02	0.026
Trapezius	0.36 (0.06; 0.75) 0.42 ± 0.36	0.90 (0.28; 1.00) 0.67 ± 0.41	<0.001	0.35 (0.00; 0.78) 0.37 ± 0.37	0.29
Levator scapulae	0.49 (0.16; 0.81) 0.47 ± 0.34	1.00 (0.67; 1.00) 0.77 ± 0.38	<0.001	0.73 (0.41; 0.91) 0.61 ± 0.34	0.026
Semispinalis capitis	0.00 (0.00; 0.13) 0.12 ± 0.23	0.57 (0.0; 1.00) 0.51 ± 0.44	<0.001	0.0 (0.0; 0.0) 0.10 ± 0.26	0.12
Longissimus capitis	0.00 (0.00; 0.00) 0.00 ± 0.00	0.0 (0.00; 0.02) 0.10 ± 0.23	<0.001	0.0 (0.0; 0.0) 0.00 ± 0.00	1
Scalenus anterior	0.00 (0.00; 0.00) 0.00 ± 0.03	0.0 (0.00; 0.00) 0.07 ± 0.20	0.002	0.0 (0.0; 0.0) 0.00 ± 0.00	0.28
Scalenus medius	0.00 (0.00; 0.00) 0.03 ± 0.11	0.0 (0.00; 0.00) 0.06 ± 0.18	0.39	0.0 (0.0; 0.0) 0.04 ± 0.16	0.42
Obliquus capitis anterior	0.00 (0.00; 0.00) 0.00 ± 0.01	0.64 (0.00; 1.00) 0.56 ± 0.46	<0.001	0.0 (0.0; 0.0) 0.00 ± 0.01	0.60
Rectus capitis major	0.00 (0.00; 0.00) 0.00 ± 0.00	0.00 (0.00; 0.00) 0.01 ± 0.08	0.50	0.0 (0.0; 0.0) 0.00 ± 0.00	1
Semispinalis cervicis	0.0 (0.0; 0.0) 0.01 ± 0.06	0.00 (0.00; 0.14) 0.15 ± 0.30	<0.001	0.0 (0.0; 0.0) 0.00 ± 0.03	0.85

Frequency of injection cycles is calculated at the subject level for each muscle. It is the ratio between the number of injection cycles in the considered muscle and the total number of injection cycles. Results are presented as the median (Q1; Q3) and mean ± standard deviation. Q1: first quartile; Q3: third quartile; US: ultrasound. * Comparison between non-guided and US-guided data within the US group (paired Wilcoxon test). ** Comparison of the No US group with non-guided injections in the US group (Mann–Whitney test).

**Table 5:** Doses of botulinum toxin and number of injected muscles (per injection cycle); abo A:ona A 2.5:1. The US group denotes the patients for whom two techniques of injection were used (at least three non-guided injection cycles followed by at least three US-guided injection cycles). The No US group denotes the patients for whom US guidance was never used (at least six non-guided injection cycles).

Muscle	US Group			No US Group	
	Non-Guided Injections	US-Guided Injections	*p* **		*p* ***
Sternocleidomastoid	50.0 (30.0; 72.0) 53.53 ± 34.46	40.0 (30.0; 60.0) 43.30 ± 24.55	0.004	50.0 (40.0; 70.0) 56.54 ± 34.45	0.31
Splenius capitis	71.0 (50.0; 100.0) 81.20 ± 44.56	40.0 (8.0; 80.0) 45.93 ± 36.16	<10^−3^	80.0 (60.0; 100.0) 90.50 ± 47.53	0.037
Trapezius	0.0 (0.0; 30.0) 16.05 ± 24.96	32.0 (0.0; 40.0) 29.91 ± 24.00	0.002	0.0 (0.0; 30.0) 14.72 ± 24.80	0.59
Levator scapulae	0.0 (0.0; 40.0) 21.54 ± 28.97	40.0 (20.0; 56.0) 36.69 ± 25.94	<10^−3^	30.0 (0.0; 40.0) 26.45 ± 26.89	0.016
Total dose	200.0 (150.0; 240.0) 206.72 ± 85.75	226.07 (160.0; 293.3) 261.68 ± 107.42	0.03	180.0 (140.0; 220.0) 220.75 ± 87.40	0.06
Mean dose per muscle *	60.0 (46.0; 76.7) 63.22 ± 26.96	45.2 (36.7; 58.0) 47.86 ± 16.6	<10^−3^	60.0 (48.0; 72.0) 63.48 ± 26.35	0.79
Number of target muscles per cycle	3.0 (3.0; 4.0) 3.55 ± 1.5	5.0 (4.0; 7.0) 5.62 ± 1.7	<10^−3^	4.0 (3.0; 4.0) 3.68 ± 1.3	0.16

Results are presented as median (Q1; Q3) and mean ± standard deviation. abo A: abobotulinumtoxin A; ona A: onabotulinumtoxin A; Q1: first quartile; Q3: third quartile; US: ultrasound. * Total dose divided by the number of injected muscles. ** Comparison between non-guided and US-guided data within the US group (linear mixed model). *** Comparison of the No US group with non-guided injections in the US group (linear mixed model).

**Table 6:** First and last BoNT doses according to the injection method (conversion ratio abo A:ona A 2.5:1). The US group denotes the patients for whom two techniques of injection were used (at least three non-guided injection cycles followed by at least three US-guided injection cycles). The No US group denotes the patients for whom US guidance was never used (at least six non-guided injection cycles).

Muscle	US Group					No US Group		
	First Dose (Non-Guided)	Last Dose (Non-Guided)	Last Dose (Guided)	*p* *	*p* **	First Dose	Last Dose	*p* ***
Sternocleidomastoid	42.5 (30.0; 50.0) 39.64 ± 16.30	50.0 (40.0; 80.0) 57.51 ± 31.46	40.0 (31.0; 50.0) 42.41 ± 20.15	<0.001	<0.001	40.0 (30.0; 50.0) 40.91 ± 14.25	50.0 (40.0; 60.0) 55.95 ± 30.57	<0.001
Splenius capitis	50.0 (40.0; 60.0) 50.20 ± 17.28	70.0 (50.0; 100.0) 83.09 ± 46.03	48.0 (32.0; 70.0) 53.01 ± 27.24	<0.001	<0.001	50.0 (50.0; 60.0) 58.43 ± 21.15	80.0 (60.0; 100.0) 84.36 ± 39.10	<0.001
Trapezius	40.0 (30.0; 50.0) 37.65 ± 13.40	40.0 (30.0; 50.0) 45.49 ± 22.71	40.0 (30.0; 40.0) 38.38 ± 16.71	0.030	0.004	40. (40.0; 50.0) 48.00 ± 19.24	40.0 (38.0; 50.0) 47.67 ± 25.78	0.75
Levator scapulae	30.0 (30.0; 48.0) 35.96 ± 16.23	40.0 (30.0; 50.0) 44.79 ± 16.86	40.0 (32.0; 58.0) 43.36 ± 16.21	0.001	0.23	30.0 (25.0; 30.0) 26.67+/- 5.77	40.0 (30.0; 53.0) 45.31 ± 18.30	0.50
Total dose	100.0 (76.25; 120.0) 103.20 ± 47.95	230.0 (182.5; 287.0) 241.59 ± 91.77	222.0 (162.0; 300.0) 245.33 ± 107.6	<0.001	0.54	100.0 (80.0; 132.5) 113.13 ± 52.06	235.0 (170.0; 297.0) 239.27 ± 86.81	0.001
Number of target muscles	2.0 (2.0; 3.0) 2.44 ± 1.12	4.0 (3.0; 5.75) 4.56 ± 1.79	6.0 (5.0; 7.0) 5.88 ± 1.86	<0.001	<0.001	2.0 (2.0; 3.0) 2.44 ± 0.87	4.0 (3.75; 5.0) 4.44 ± 1.53	0.001

Results are presented as the median (Q1; Q3) and mean ± standard deviation. Q1 first quartile; Q3 third quartile; US: ultrasound. Dose comparisons were performed using the paired Wilcoxon’s test. * US group: first dose non-guided vs. last dose non-guided. ** US group: last dose non-guided vs. last dose US-guided. *** No US group: first dose vs. last dose.

**Table 7:** Comparisons of botulinum toxin dose change over time (and number of injected muscles) according to the technique of guidance (conversion ratio abo A:ona A 2.5:1). The US group denotes the patients for whom two techniques of injection were used (at least three non-guided injection cycles followed by at least three US-guided injection cycles). The No US group denotes the patients for whom US guidance was never used (at least six non-guided injection cycles).

Muscle	US Group: Non-Guided vs. US-Guided Injections *	US Group Non-Guided Injections vs. No US Group *
Sternocleidomastoid	NA	NA
Splenius capitis	<10^−3^	0.47
Trapezius	0.67	0.94
Levator scapulae	0.0018	0.57
Total dose	<10^−3^	0.068
Mean dose per muscle **	<10^−3^	0.064
Number of injected muscles	0.15	0.82

US: ultrasound; NA: not applicable (no evolution over time has been shown; for more detail, see Table S3 in the Supplementary Materials). * *p*-values for the existence of a different evolution in BoNT dose (and number of injected muscles) according to the technique of guidance. Change over time in injected dose was modelized using a linear mixed model for repeated measurements with a linear and quadratic time effect as well as the interactions between these effects of time and the type of guidance. ** Total dose divided by the number of injected muscles.

## Data Availability

The data presented in this study are available in this article and Supplementary Materials.

## Supplementary Materials

The following supporting information can be downloaded at https://www.mdpi.com/article/10.3390/toxins16100439/s1, Table S1. Doses of botulinum toxin and number of injected muscles (per injection cycle); abo A:ona A 3:1. The US group denotes the patients for whom two techniques of injection were used (at least three non-guided injection cycles followed by at least three US-guided injection cycles). The No US group denotes the patients for whom US guidance was never used (at least six non-guided injection cycles). Table S2. First and last BoNT doses according to the injection method (conversion ratio abo A:ona A 3:1). Table S3. Test for change over time in the botulinum toxin dose (and number of injected muscles) according to the technique of guidance (conversion ratio abo A:ona A 2.5:1). The US group denotes the patients for whom two techniques of injection were used (at least three non-guided injection cycles followed by at least three US-guided injection cycles). The No US group denotes the patients for whom US guidance was never used (at least six non-guided injection cycles). Table S4. Comparisons of botulinum toxin dose change over time (and number of injected muscles) according to the technique of guidance (conversion ratio abo A:ona A 3.0:1). The US group denotes the patients for whom two techniques of injection were used (at least three non-guided injection cycles followed by at least three US-guided injection cycles). The No US group denotes the patients for whom US guidance was never used (at least six non-guided injection cycles). Table S5. Test for change over time in the botulinum toxin dose (and number of injected muscles) according to the technique of guidance (conversion ratio abo A:ona A 3.0:1). The US group denotes the patients for whom two techniques of injection were used (at least three non-guided injection cycles followed by at least three US-guided injection cycles). The No US group denotes the patients for whom US guidance was never used (at least six non-guided injection cycles).
