# Supplementary material for: Doses of Botulinum Toxin in Cervical Dystonia: Does Ultrasound Guidance Change Injection Practices?
Source: Toxins (Basel). 2024 Oct 11;16(10):439. doi: 10.3390/toxins16100439 (PMC11511466; doi:10.3390/toxins16100439)
Supplement: Supplementary file 1 [file toxins-16-00439-s001.zip › toxins-3216300-supplementary.pdf]

Supplementary Materials

Table S1. Doses of botulinum toxin and number of injected muscles (per injection cycle); abo A:ona A 3:1. The "US group" denotes the patients for whom two techniques of injection were used (at least three non-guided injection cycles followed by at least three US-guided injection cycles). The "No US group" denotes the patients for whom US guidance was never used (at least six non-guided injection cycles).

Muscle	US group			No US group	
	Non-guided injections	US-guided injections	p**		p***
Sternocleidomastoid	50.0 (30.0;70.0)<br>52.02 +/-32.57	40.0 (26.7;53.3)<br>38.59 +/-22.35	$< 10^{-3}$	50.0 (40.0;70.0)<br>55.08+/-31.30	0.30
Splenius capitis	70.0 (50.0;100.0)<br>78.37 +/-39/57	40.0 (6.7;66.7)<br>41.71+/-33.64	$< 10^{-3}$	80.0 (60.0;100.0)<br>87.95+/-42.23	0.037
Trapezius	0.0 (0.0;30.0)<br>15.77+/-24.61	30.0 (0.0;40.0)<br>25.94+/-20.40	0.015	0.0 (0.0;30.0)<br>14.32+/-24.31	0.58
Levator scapulae	0.0 (0.0;40.0)<br>20.87 +/-28.28	33.3 (16.7;50.0)<br>32.37+/-22.79	$< 10^{-3}$	30.0 (0.0;40.0)<br>25.85+/- 26.34	0.015
Total dose	200 (150.0;240.0)<br>200.70+/-78.06	226.67 (160.0;193.3)<br>232.22+/-93.02	<.001	180.0 (140.0;220.0)<br>214.64+/-78.78	0.13
Mean dose per muscle*	58.3 (45.0;75.0)<br>61.42+/-24.66	40.7 (32.5;50.0)<br>42.23+/-14.22	$< 10^{-3}$	60.0 (46.7;70.0)<br>61.86+/-23.26	$< 10^{-4}$
Number of target muscles per cycle	3.0 (3.0;4.0)<br>3.55 +/1.50	6.0 (4.0;7.0)<br>5.64+/-1.80	$< 10^{-3}$	4.0 (3.0;4.0)<br>3.68+/-1.38	0.16

Results are presented as median (Q1;Q3) and mean ± standard deviation. abo A: abobotulinumtoxin A; ona A: onabotulinumtoxin A; Q1: first quartile; Q3: third quartile; US: ultrasound.

*Total dose divided by the number of injected muscles.

**Comparison between non-guided and US-guided data within the "US group" (linear mixed model).

***Comparison of the "No US group" with non-guided injections in the "US group" (linear mixed model).

Table S2. First and last BoNT dose according to the injection method (conversion ratio abo A:ona A 3.0:1). The "US group" denotes the patients for whom two techniques of injection were used (at least three non-guided injection cycles followed by at least three US-guided injection cycles). The "No US group" denotes the patients for whom US guidance was never used (at least six non-guided injection cycles).

Muscle	US group					No US group		
	First dose (non-guided)	Last dose (non-guided)	Last dose (guided)	p*	p**	First dose	Last dose	p***
Sternocleidomastoid	45.0 (30.0;50.0)<br>40.71+/-14.89	50.0 (38.33;71.25)<br>54.50+/-28.48	40.0 (26.67;50.0)<br>38.71+/-19.50	<0.001	<0.001	40.0 (30.0;50.0)<br>40.39+/-13.26	48.33 (33.33;60.0)<br>52.70+/-28.31	0.002
Splenius capitis	50.0 (40.0;60.0)<br>51.03+/-18.05	70.0 (50.0;100.0)<br>78.91+/-39.83	40.0 (30.0;66.67)<br>47.37+/-25.13	<0.001	<0.001	50.0 (50.0;60.0)<br>57.46+/-18.62	80.0 (60.0;100.0)<br>81.27+/-34.72	<0.001
Trapezius	33.33 (30.0;50.0)<br>37.17+/-13.51	40.0 (30.0;50.0)<br>42.74+/-18.86	33.33 (26.67;40.0)<br>34.59+/-14.22	0.07	0.007	40.0 (40.0;50.0)<br>48.00+/-19.24	40.0 (33.33;50.0)<br>46.23+/-24.34	0.75
Levator scapulae	30.0 (30.0;40.0)<br>35.39+/-14.91	40.0 (30.0;50.0)<br>42.99+/-15.90	40.0 (30.0;50.0)<br>43.11+/-32.76	0.002	0.09	30.0 (20.0;30.0)<br>30.00+/-12.25	40.0 (30.0;50.0)<br>43.46+/-17.30	0.25
Total dose	100.0 (76.25;130.0)<br>101.96+/-45.24	225.0 (180.0;280.0)<br>230.22+/-78.90	191.67 (145.42;290.0)<br>217.07+/-94.93	<0.001	0.07	100.0 (80.0;132.5)<br>111.39+/-48.22	230.0 (158.33;282.5)<br>225.45+/-84.00	<0.001
Number of target muscles	2.0 (2.0;3.0)<br>2.43+/-1.12	4.0 (3.0;5.0)<br>4.56+/-1.76	6.0 (5.0;7.0)<br>5.85+/-1.91	<0.001	<0.001	2.0 (2.0;3.0)<br>2.44+/-0.87	4.0 (3.0;5.0)<br>4.33+/-1.59	<0.001

Results are presented as median (Q1;Q3) and mean ± standard deviation. Q1 first quartile; Q3 third quartile; US: ultrasound.
Dose comparisons were performed using the paired Wilcoxon's test.
*"US group": first dose non-guided vs last dose non-guided.
**"US group": last dose non-guided vs last dose US-guided.
***"No US group": first dose vs last dose.

Table S3. Test for change over time in the botulinum toxin dose (and number of injected muscles) according to the technique of guidance (conversion ratio abo A:ona A 2.5:1). The "US group" denotes the patients for whom two techniques of injection were used (at least three non-guided injection cycles followed by at least three US-guided injection cycles). The "No US group" denotes the patients for whom US guidance was never used (at least six non-guided injection cycles).

Muscle	"US group" Non-guided*	"US group" US-guided*	"No US group"*
Sternocleidomastoid	Linear B=0.06 (p=0.25) Quadratic B=0.00 (p=0.87)	Linear B=0.12 (p=0.21) Quadratic B=-0.02 (p=0.36)	Linear B=0.03 (p=0.35) Quadratic B=-0.01 (p=0.21)
Splenius capitis	Linear B=0.17 ($p< 10^{-3}$) Quadratic B=-0.01 ($p< 10^{-3}$)	Linear B=-0.12 (p=0.31) Quadratic B=-0.01 (p=0.78)	Linear B=0.12 (p=0.0033) Quadratic B=-0.01 (p=0.0021)
Trapezius	Linear B=0.25 (p=0.0042) Quadratic B=-0.02 (p=0.0078)	Linear B=0.33 (p=0.021) Quadratic B=-0.04 (p=0.15)	Linear B=0.26 (p=0.001) Quadratic B=-0.02 (p=0.010)
Levator scapulae	Linear B=0.51 ($p< 10^{-3}$) Quadratic B=-0.04 ($p< 10^{-3}$)	Linear B=0.27 (p=0.037) Quadratic B=-0.06 (p=0.038)	Linear B=0.48 ($p< 10^{-3}$) Quadratic B=-0.03 ($p< 10^{-3}$)
Total dose	Linear B=0.18 ($p< 10^{-3}$) Quadratic B=-0.01 ($p< 10^{-3}$)	Linear B=0.05 (p=0.22) Quadratic B=-0.01 (p=0.041)	Linear B=0.10 ($p< 10^{-3}$) Quadratic B=-0.01 ($p< 10^{-3}$)
Mean dose per muscle**	Linear B=0.06 ($p< 10^{-3}$) Quadratic B=-0.01 ($p< 10^{-3}$)	Linear B=0.06 (p=0.015) Quadratic B=-0.01 (p=0.57)	Linear B=0.02 (p=0.19) Quadratic B=-0.01 (p=0.81)
Number of injected muscles	Linear B=0.11 ($p< 10^{-3}$) Quadratic B=-0.01 ($p< 10^{-3}$)	Linear B=0.08 ($p< 10^{-3}$) Quadratic B=-0.01 (p=0.041)	Linear B=0.09 ($p< 10^{-3}$) Quadratic B=-0.01 ($p< 10^{-3}$)

US: ultrasound; B: coefficient of time effect (linear and quadratic) estimated by the mixed model for longitudinal data.

*: p values for a significant evolution over time in the botulinum toxin dose dose (and the number of injected muscles) according to the technique of guidance. Change over time in injected dose (and number of injected muscles) is modelized using a linear mixed model for repeated measurements with a linear and quadratic time effect. A significant quadratic effect indicates a curvilinear evolution.

**Total dose divided by the number of injected muscles.

Table S4. Comparisons of botulinum toxin dose change over time (and number of injected muscles) according to the technique of guidance (conversion ratio abo A:ona A 3.0:1). The "US group" denotes the patients for whom two techniques of injection were used (at least three non-guided injection cycles followed by at least three US-guided injection cycles). The "No US group" denotes the patients for whom US guidance was never used (at least six non-guided injection cycles).

Muscle	"US group": non-guided vs US-guided injections*	"US group" non-guided injections vs "No US group"*
Sternocleidomastoid	NA	NA
Splenius capitis	$< 10^{-3}$	0.48
Trapezius	0.66	0.94
Levator scapulae	0.0017	0.58
Total dose	$< 10^{-3}$	0.078
Mean dose per muscle**	$< 10^{-3}$	0.082
Number of injected muscles	0.092	0.82

US: ultrasound; NA: not applicable (no evolution over time has been shown; for more detail, see Table E in Supplementary material).
*: p values for the existence of a different evolution in BoNT dose (and number of injected muscles) according to the technique of guidance. Change over time in injected dose was modelized using a linear mixed model for repeated measurements with a linear and quadratic time effect as well as the interactions between these effects of time and the type of guidance.
**Total dose divided by the number of injected muscles.

Table S5. Test for change over time in the botulinum toxin dose (and number of injected muscles) according to the technique of guidance (conversion ratio abo A:ona A 3.0:1). The "US group" denotes the patients for whom two techniques of injection were used (at least three non-guided injection cycles followed by at least three US-guided injection cycles). The "No US group" denotes the patients for whom US guidance was never used (at least six non-guided injection cycles).

Muscle	"US group": Non-guided*	"US group": US-guided*	"No US group"*
Sternocleidomastoid	Linear B=0.05 (p=0.30) Quadratic B=0.01 (p=0.81)	Linear B=0.11 (p=0.21) Quadratic B=-0.02 (p=0.35)	Linear B=0.03 (p=0.38) Quadratic B=-0.01 (p=0.21)
Splenius capitis	Linear B=0.17 (< 10^{-3}) Quadratic B=-0.01 (p< 10^{-3})	Linear B=-0.1(p=0.30) Quadratic B=-0.01 (p=0.78)	Linear B=0.11 (p=0.0037) Quadratic B=-0.01 (p=0.0021)
Trapezius	Linear B=0.24 (p=0.0045) Quadratic B=-0.21 (p=0.0083)	Linear B=0.32 (p=0.020) Quadratic B=-0.04 (p=0.14)	Linear B=0.26 (p< 10^{-3}) Quadratic B=-0.02 (p=0.010)
Levator scapulae	Linear B=0.50 (p< 10^{-3}) Quadratic B=-0.04 (p< 10^{-3})	Linear B=0.26 (p=0.037) Quadratic B=-0.06 (p=0.038)	Linear B=0.47 (p< 10^{-3}) Quadratic B=-0.03 (p< 10^{-3})
Total dose	Linear B=0.18 (p< 10^{-3}) Quadratic B=-0.01 (p< 10^{-3})	Linear B=0.05 (p=0.25) Quadratic B=-0.02 (p=0.055)	Linear B=0.10 (p< 10^{-3}) Quadratic B=-0.01 (p< 10^{-3})
Mean dose per muscle**	Linear B=0.06 (p< 10^{-3}) Quadratic B=-0.01 (p< 10^{-3})	Linear B=-0.07 (p=0.0029) Quadratic B=0.01 (p=0.20)	Linear B=0.01 (p=0.30) Quadratic B=-0.01 (p=0.10)
Number of injected muscles	Linear B=0.11 (p< 10^{-3}) Quadratic B=-0.01 (p< 10^{-3})	Linear B=0.09 (p< 10^{-3}) Quadratic B=-0.01 (p=0.015)	Linear B=0.09 (p< 10^{-3}) Quadratic B=-0.01 (p< 10^{-3})

US: ultrasound; B: coefficient of time effect (linear and quadratic) estimated by the mixed model for longitudinal data.
*: p values for a significant evolution over time in the botulinum toxin dose dose (and the number of injected muscles) according to the technique of guidance. Change over time in injected dose (and number of injected muscles) is modelized using a linear mixed model for repeated measurements with a linear and quadratic time effect. A significant quadratic effect indicates a curvilinear evolution.
**Total dose divided by the number of injected muscles.
